# Chirped InGaAs quantum dot molecules for broadband applications

**DOI:** 10.1186/1556-276X-7-207

**Published:** 2012-04-06

**Authors:** Nirat Patanasemakul, Somsak Panyakeow, Songphol Kanjanachuchai

**Affiliations:** 1 Department of Electrical Engineering, Faculty of Engineering, Semiconductor Device Research Laboratory (Nanotec Center of Excellence), Chulalongkorn University, Bangkok 10330, Thailand

**Keywords:** Quantum dot molecules, InAs, InGaAs, Chirp, Broadband, Photoluminescence

## Abstract

Lateral InGaAs quantum dot molecules (QDMs) formed by partial-cap and regrowth technique exhibit two ground-state (GS) peaks controllable via the thicknesses of InAs seed quantum dots (*x*), GaAs cap (*y*), and InAs regrowth (*z*). By adjusting *x*/*y*/*z *in a stacked QDM bilayer, the GS peaks from the two layers can be offset to straddle, stagger, or join up with each other, resulting in multi-GS or broadband spectra. A non-optimized QDM bilayer with a 170-meV full-width at half-maximum is demonstrated. The temperature dependencies of the emission peak energies and intensities from the chirped QDM bilayers are well explained by Varshni's equation and thermal activation of carriers out of constituent quantum dots.

## Background

Narrow- and broadband optoelectronic devices in the 0.9- to 1.55-μm range benefit greatly from self-assembled InGaAs quantum dots (QDs). The conflicting narrow and broad bandwidth requirements lead to different procedures during the growth of QD active layers. While processes for narrowband devices such as lasers seek to minimize the inhomogeneities intrinsic to Stranski-Krastanow (SK) QDs [1], those for broadband devices such as superluminescent diodes (SLDs) seek to maximize [2-4] and enhance them by adopting a chirped structure [5] where several QD layers are stacked in a wider-gap matrix and by varying the properties of the individual QD layers, the matrix, or both [6-9]. The number of stacks in strained heteroepitaxy, however, should be kept low to minimize accumulated strain [10]. To reduce the stack number in chirped QD structures without compromising bandwidth, the QDs can be replaced by certain types of lateral quantum dot molecules (QDMs). QDMs can be broadly described as a system of coupled QDs where QDs are spaced closely vertically, in the growth direction, or laterally, in the growth plane [11]. Vertical QDMs have been a subject of intense interest since the demonstrations of QD coupling [12] and entanglement of quantum states [13] which form the foundation of quantum computation [14]. Vertical coupling, however, cannot be tuned postgrowth in contrast to lateral QDMs whose tunnel barriers can be tuned electrically using surface or side gates and thus extend the scope of quantum interactions that can be studied. In contrast to their potentials as coupled quantum systems, lateral QDMs are often overlooked as device active layers mainly because self-assembly of lateral QDMs is more involved and partly because optical properties of QD and QDM ensembles do not differ significantly. We recently developed a capping and regrowth procedure [15] to form lateral QDMs where each molecule comprises a large, central QD (cQD) and several small, satellite QDs (sQDs). Our QDMs exhibit structural *and *optical bimodalities [16] and have been incorporated in solar cells [17]. Despite many reports of other lateral QDM growth strategies [18-21], a device structure designed specifically to take advantage of intrinsic QDM characteristics has been lacking. In the simplest case of two QDs per molecule, however, devices such as photodetectors have been reported [22].

In this letter, we propose and demonstrate an alternative chirped structure whose active layer is a lateral InGaAs QDM bilayer. The proposed structure departs from conventional chirped QDs and quantum wells but offers superior broadband luminescence or response in the near-infrared (NIR) region with only a few QDM layers grown under standard conditions. The temperature-dependent photoluminescence of the chirped QDM structures can be well explained using multi-Gaussian distributions, each with characteristic activation energy and fitting parameters related to escape channels for carriers in cQDs and sQDs.

## Methods

All InGaAs QDM samples are grown on (001)-GaAs substrates by solid source molecular beam epitaxy (MBE). After oxide desorption at 610°C, 300-nm GaAs is grown at 580°C, followed by a QDM layer and 100-nm GaAs. Growth stops here for single QDM layer structures. For chirped QDM bilayer structures, growth continues with an additional QDM layer and a final 100- nm GaAs. The decoupled lower and upper QDM layers are different. A typical QDM layer is formed via the partial-cap and regrowth technique [15] where *x *monolayers (MLs) of InAs seed QDs are grown at 500°C, then capped by *y*-ML GaAs at 470°C after which nanoholes are formed and used as a template for regrowth of *z*-ML InAs QDs at 470°C. After regrowth, GaAs capping proceeds at 470°C for the first 10 nm and at 500°C for the remaining 90 nm. Each QDM layer can be controlled by varying *x*, *y*, and *z*; the constituent nanostructure is thus referred to hereafter as *x*/*y*/*z *QDMs. The formation of QDs, nanoholes, and QDMs can be observed *in situ *via reflection high-energy electron diffraction patterns, while the morphologies of layers of interest are probed *ex situ *by atomic force microscopy (AFM). Series of samples where either a single QDM layer or chirped QDM bilayer constitutes the active layer are grown and characterized by photoluminescence (PL). In all samples, *x *= 1.9 or 2 ML, *y *= 6 to 25 ML, and *z *= 1.4 to 2 ML. Samples are mounted in a closed-cycle He cryostat and excited by a 476.5-nm Ar^+ ^laser. The PL signals are dispersed in a 1-m spectrometer and collected by a cooled InGaAs detector.

## Results and discussion

The morphology and optical properties of single QDM layers will first be discussed, followed by the optical properties of the chirped QDM bilayers, their temperature dependencies, and the physical mechanisms that govern them.

### Single QDMs

**Figure 1:**
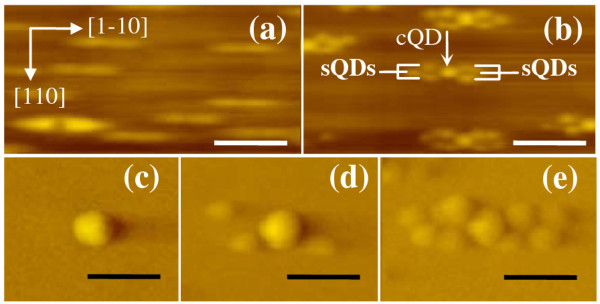
**AFM images of uncapped nanoholes and QDMs at various stages**. (**a**) Nanoholes, (**b**) fully-developed QDMs, (**c**) early-stage QDMs where sQDs are absent, (**d**) partially-developed QDMs where some sQDs are present around cQD, and (**e**) fully-developed QDMs. The white (black) scale bars are 250 nm (125 nm).

**Figure 2:**
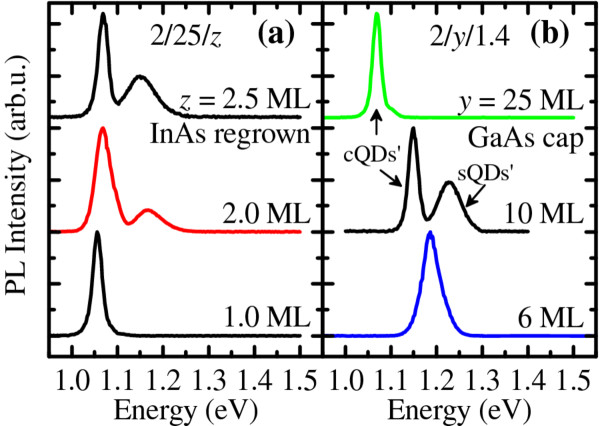
**Low-temperature PL spectra of single QDM layers**. 20-K PL spectra of (**a**) 2/25/*z *QDMs with *z *= 1, 2, or 2.5 ML and (**b**) 2/*y*/1.4 QDMs with *y *= 6, 10, or 25 ML. Spectra are offset and rescaled for clarity.

**Figure 3:**
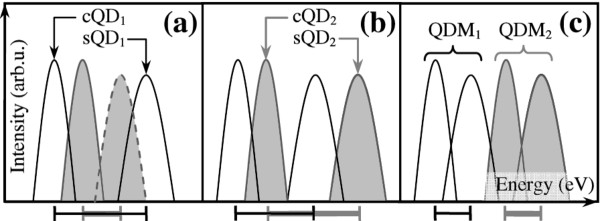
**Schematic spectra of individual luminescent peaks from cQDs and sQDs in chirped QDM bilayers**. Spectra of (**a**) straddled or type-I, (**b**) staggered or type-II, and (**c**) broken-gap or type-III chirped QDM bilayers. Subscripts 1 and 2 denote the lower and upper QDM layers, respectively. The dashed curve in (a) indicates optional sQD_2 _peak. The black (gray) bar marks the spectral range between the cQD and sQD peaks of QDM_1 _(QDM_2_).

A typical morphology of 2/6/0 nanoholes (2-ML InAs seed QDs capped by 6-ML GaAs with no regrowth) is shown in the AFM image in Figure 1a. All the AFM images are not deconvoluted; apparent lateral dimensions are thus greater than actual dimensions by the degree set by the scanning tip's radius. Vertical dimensions are however unaffected. Each nanohole has a shallow dimple approximately 0.4 to 1 nm in depth with mounds elongated in the [1-10] direction. The resulting shape is routinely observed in As_4_, but not in As_2 _epitaxy [23]. After 1.4-ML InAs regrowth, QDMs are formed as shown in Figure 1b. The average number of sQDs for each QDM increases with regrowth thickness *z *and can vary from 0 in Figure 1c to 3 in Figure 1d and to 6 or more in Figure 1e.

The 20-K PL spectra of two sample series with a single QDM layer in each sample are shown in Figure 2a for 2/25/*z* QDMs where *z* = 1, 2, or 2.5 ML and in Figure 2b for 2/*y*/1.4 QDMs where *y* = 6, 10, or 25 ML. Figure 2a shows that as the regrowth thickness for 2/25/*z* QDMs increases from 1 to 2 ML, the number of ground-state (GS) peaks increases from 1 to 2 which correlates with surface morphology before and after the nucleation of sQDs, respectively. cQDs are present in both cases. The low (high) energy peak is thus assigned to emissions from cQDs (sQDs). As *z* increases to 2.5 ML, the cQD peak remains almost unaltered while the sQD peak red-shifts and broadens slightly. The cQD peak remains almost unaltered because the nanoholes are being filled and saturating, while the sQD peak red-shifts and broadens because the resulting greater material availability makes sQDs grow proportionately. Similar double GS peaks in lateral QDMs have been reported [23], and their temperature dependencies have been discussed in detail elsewhere [24].

Figure 2b shows, from bottom to top, that for a fixed 1.4-ML InAs regrowth, the PL spectra exhibit two unresolved peaks, two separate peaks, and one peak as the GaAs cap thickens from *y *= 6 to 10 and 25 ML, respectively. This is due to the difference in average nanohole depth which increases with *y *[25]. Shallow holes (*y *= 6 ML) are easily filled: at 1.4-ML regrowth, QDMs are fully formed and the average sizes of sQDs and cQDs are similar. Their GSs thus significantly overlap as seen in the bottom curve. A QDM under this condition is depicted in Figure 1e. Deep holes (*y *= 25 ML) are not easily filled: for the same 1.4-ML regrowth, QDMs are not fully formed since nanoholes have yet to be saturated - cQDs exist but sQDs are absent, as depicted in Figure 1c, resulting in a single PL peak in the top curve. At *y *= 10 ML, the situation is intermediate between the two cases: sQDs exist but with the average size (and corresponding PL peak energy) noticeably smaller (higher) than cQDs as seen in Figure 1d (middle curve of Figure 2b).

Lateral QDMs spectra thus exhibit three basic PL characteristics: single peak, non-overlapping double peak, and overlapping double peak - controllable via *z*. In chirped QDM bilayer structures, two of the three characteristics can be combined to yield broadband or multi-GS emissions which can serve as active materials for SLDs [26] or dual-wavelength QD lasers [27], respectively.

### Chirped QDM bilayers

Depending on the growth parameters of the lower (QDM_1_) and upper (QDM_2_) dot layers, the GS peaks from the two layers can be offset in a straddled (type I), staggered (II), or broken-gap (III) configuration as schematically shown in Figure 3a,b,c, respectively. (The nomenclatures are adopted from the three basic types of band offsets at hetero-interfaces [28].)

**Figure 4:**
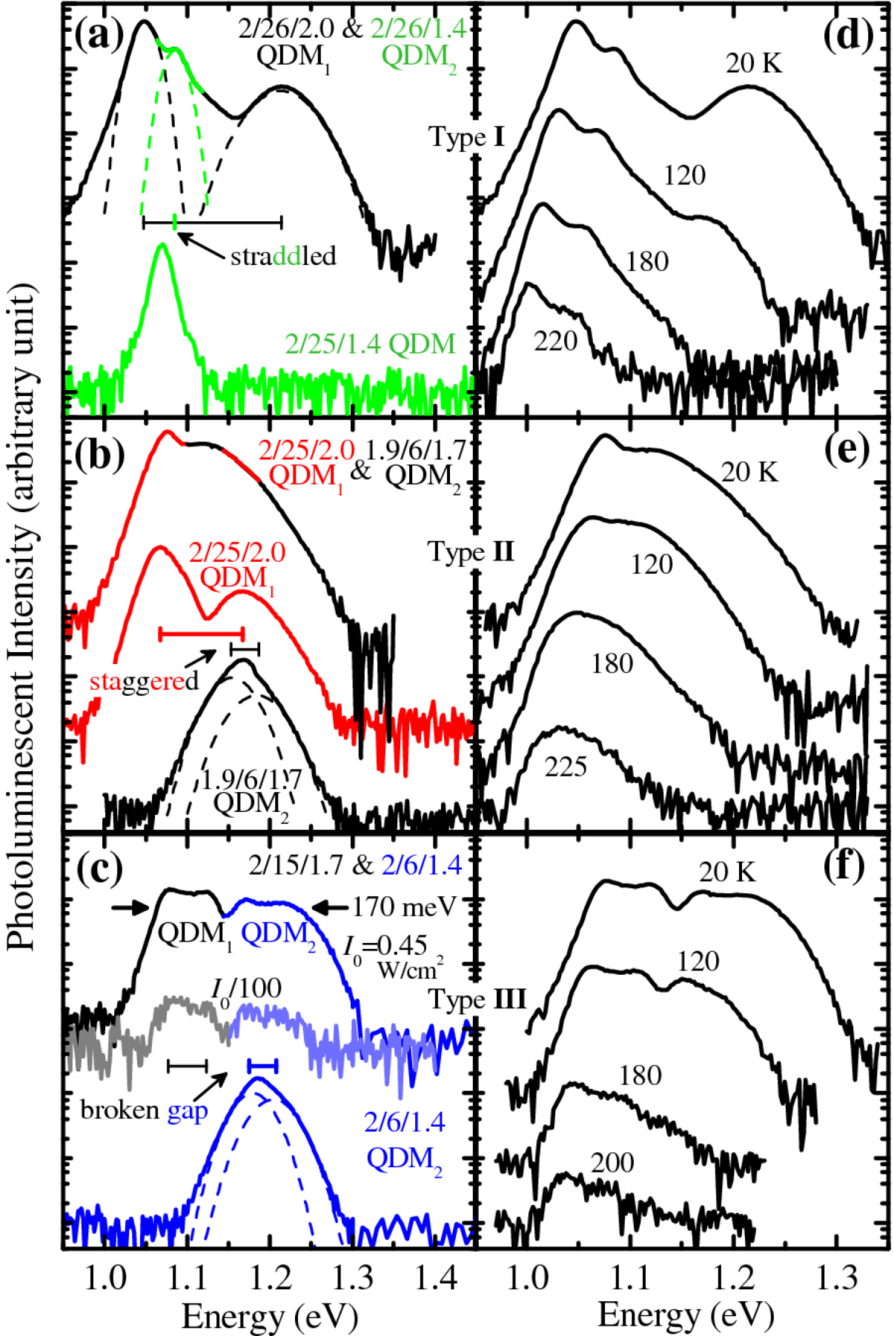
**Measured 20-K PL and temperature-dependent PL spectra of chirped QDM bilayers**. 20-K PL spectra of samples containing a single QDM layer or chirped QDM bilayer as active layers: (**a**) single 2/25/1.4 QDM reference (lower plot), and chirped 2/26/2.0 QDM_1 _bottom layer and 2/26/1.4 QDM_2 _top layer (upper); (**b**) single 1.9/6/1.7 QDM_2 _(lower), single 2/25/2.0 QDM_1 _(middle), and chirped QDM_1_/QDM_2 _bilayer (upper); and (**c **) single 2/6/1.4 QDM_2 _(lower plot), and chirped 2/15/1.7 QDM_1 _and 2/6/1.4 QDM_2 _bilayer under nominal (top) and reduced excitations (middle). Temperature-dependent PL spectra of the chirped QDM bilayers in (a), (b) and (c) are shown in (**d**), (**e**), and (**f**), respectively. Dashed lines in (a to c) are multiple Gaussian function fits. Spectra are offset for clarity.

Type-I chirps require emissions from one active layer to be straddled or confined by the other. This is demonstrated in a 2/26/2 QDM_1 _and 2/26/1.4 QDM_2 _bilayer stack. The 20-K PL spectra of the bilayer sample is shown (upper curve) in Figure 4a, together with those of a single 2/25/1.4 QDM layer reference (lower). The latter emits one cQD peak at 1.070 eV, while the former emits three resolved GS peaks at 1.048, 1.084, and 1.214 eV assigned to cQD_1_, cQD_2_, and sQD_1_, respectively. QDM_1_'s GS peaks - cQD_1 _and sQD_1 _- are as expected: they red-shift from the smaller 1.8/25/1.2 QDMs whose peaks were previously reported at 1.075 eV for cQDs and 1.240 eV for sQDs [16]. By regrowing below nanohole saturation point, the upper 2/26/1.4 QDM_2 _layer comprises mainly cQDs which are smaller and thus emit a peak energy higher than those of the 2/26/2 QDM_1 _layer and of the reference 2/25/1.4 QDM layer. The cQD2 peak thus appears as a hump between the cQD_1 _and sQD_1 _peaks, smoothening but not removing the cQD_1_-sQD_1 _dip between 1.05 and 1.15 eV.

Type-II chirps require the cQD_2 _peak to be between the cQD_1 _and sQD_1 _peaks, and the sQD_1 _peak to be between the cQD_2 _and sQD_2 _peaks. Figure 4b shows an example of this design with spectra from the single 1.9/6/1.7 QDM_2 _layer (bottom curve), single 2/25/2 QDM_1 _layer (middle), and QDM_1_/QDM_2 _bilayer (top) samples grown under otherwise identical conditions. The spectrum of the bilayer sample is almost a linear combination of the spectra of the two single QDM layers, indicating that the 100-nm GaAs spacer is sufficiently thick to decouple the bilayer [29]. The strong cQD_2 _peak removes the cQD_1_-sQD_1 _dip, giving a smooth spectrum.

Type-III chirps can be formed if the two QDM layers have sufficiently different capping thicknesses, to ensure that the cQD_1 _and cQD_2 _peaks are well separated, and thick regrowth, so that the sQD_1 _peak is at a lower energy than the cQD_2 _peak. This is demonstrated in a 2/15/1.7 QDM_1 _and 2/6/1.4 QDM_2 _bilayer. The 20-K PL spectra of the bilayer sample is shown (upper curve) in Figure 4c, together with those of separately grown single QDM_2 _layer reference (lower). The latter - with a misleading single peak in the linear plot in Figure 2b - can be well described by a double Gaussian function (dashed lines) corresponding to the cQD_2 _and sQD_2 _peaks which appear on the right half of the upper curves whose left half can also be described by another double Gaussian function corresponding to QDM_1 _(not shown). The four Gaussian peaks can be resolved almost down to the noise floor as seen when comparing the PL spectra under nominal (*I*_0 _= 0.45 W/cm^2^, upper curve) and reduced (*I*_0_/100, middle) excitations, confirming that all peaks are ground states. Ignoring the small dip in the middle, the full-width at half-maximum (FWHM) of this *two*-stack chirped QDM structure is 170 meV, broader than the 125 meV achieved in a *four*-stack chirped QD structure [6], yet slightly narrower than the 200 meV in a *sixty*-stack strain-compensated structure [9], illustrating the potentials of chirped QDM structures as a broadband material.

### Temperature dependencies

A broadband spectrum at low temperatures is the most important characteristic of a material destined for broadband applications, but does not necessarily translate to useful room-temperature devices. The latter require proper barrier design to minimize carrier loss by non-radiative recombination (NRR) mechanisms which can be deduced from temperature-dependent PL measurements.

The PL spectra of the three chirped QDM bilayers from the 20-K base temperature to 225 K are shown in Figure 4d for type-I, Figure 4e for type-II, and Figure 4f for type-III designs. The overall luminescence of the three types can be seen to gradually decrease as temperature increases. The spectra are discernable from the noise floors up to approximately 230 K for all structures. This does not rule out the material for room-temperature operation since cladding layers such as Al(Ga)As/GaAs superlattices can be employed to increase luminescence efficiency of the structures. The low-energy peaks slowly red-shift as the temperature increases and can be accurately modeled using Varshni's equation with bulk InAs parameters (see later). The high-energy end of the spectra is quenched at temperatures lower than the low-energy end, which is in good agreement with temperature-dependent characteristics of single QDM layers which have been explained by thermal activation of carriers out of QDMs into the wetting layer (WL) [16,24].

In order to model the temperature dependencies of the chirped QDM bilayers, a few assumptions are made. One, the bilayers are completely decoupled; the spectra of the bilayers are thus a linear combination of the spectra of individual QDM layers: QDM_1 _and QDM_2_. This is a realistic assumption since the GaAs spacer layer between the bilayer is 100-nm thick, almost twice the 200 ML determined by Xie et al. to be the thickness necessary to completely decouple stacked InAs/GaAs layers [29]. In addition, the three chirped structures are designed such that one or both of the lower QDM emission peaks are lower in energy than the upper QDM layer, minimizing reabsorption in surface-emitting applications.

Two, the temperature variation of cQDs's peak energies follow Varshni's equation with bulk InAs parameters. The cQDs nucleate inside the nanohole template dug out of seed InAs QDs and are thus expected to exhibit the same temperature dependencies as typical InAs QDs. The seed InAs QDs are grown at a low rate of 0.01 ML/s, resulting in uniform QDs with typical FWHM in the range of 28 to 40 meV. For uniform InAs/GaAs QD ensembles, the temperature dependencies of GS peaks typically follow Varshni's equation with bulk InAs parameters, while those of FWHM remain virtually constant from 20 to 300 K [30,31]. As a first approximation, the cQDs's FWHM are assumed constant.

Three, the temperature variation of sQDs's peak energies follow Varshni's equation up to about 80 K, above which they decrease at a faster rate of -1 meV/K. This assumption is based on a previous observation of single QDM layers [16] and usually referred to as a sigmoidal behavior resulting from carrier redistribution among non-uniform QD ensembles [32-35]. The FWHM of such ensembles exhibits a temperature anomaly where it decreases at an intermediate temperature and increases again to recover, or even exceed, the low-temperature value as the temperature increases towards room temperature. Only non-uniform sQDs (thin regrowth) follow the sigmoidal and anomalous behaviors; uniform sQDs (thick regrowth) are treated the same way cQDs are.

The above assumptions allow us to model the PL intensity *I *arising from the chirped QDM bilayers at various energies *E *and temperatures *T *using the expression:

$$I=\overset{4}{\underset{i=1}{\sum }}\frac{I_{i}\exp{\left(E-E_{i}\right)}^{2}/\Gamma^{2}}{1+A\exp-\left(E-E_{WL}\right)/\eta_{i}kT}$$

where *I_i _*is the normalized intensity of a PL peak at the base temperature, *E_i _*is the GS peak energy, Γ is the broadening parameter, *A *= 3 × 10^8 ^is the pre-exponential factor, *E_WL _*= 1.42 eV is the WL's energy appropriate for our structures [16], *k *is the Boltzmann constant, and *η_i _*is an ideality factor indicating the effective potential barrier felt by carriers in cQDs or sQDs of the layer of interest. The summation over *i *from 1 to 4 represents the four GS peaks of the QDM bilayer originating from the two constituent ensembles - cQDs and sQDs - of each layer. The numerator inside the summation is simply the Gaussian function describing inhomogeneity associated with SK QDs [36], while the denominator signifies the PL intensity reduction term similar in form and cause to those of quantum wells [37] which has been applied to QD ensembles with varying degrees of sophistication [34,38-41]. The only difference here is the introduction of the ideality factor *η *which varies between 1 and 2. The lower limit *η *= 1 is the ideal case which implies that electrons and holes in the QDs are lost in pairs through thermal barriers [37]. The upper limit *η *= 2 indicates that the effective barrier is half the ideal case which is possible if (1) the loss of either carrier- electrons or holes -is the rate-limiting factor in which case band offsets would dictate the effective barrier [37] or (2) multiple loss mechanisms/channels exist and are acting in parallel. Besides thermionic emissions of carriers from QDMs to NRR centers in the WL and bulk GaAs, other possible NRR centers and transport channels present in our lateral QDMs are the nanohole template, interfacial defects, and strain-induced localized states [42], and, considering the close proximity between cQD and sQDs in a QDM, may possibly involve tunneling [41]. If two independent, thermally-activated NRR channels dominate, the intensity reduction term can be accurately modeled using a double activation energy term as reported by Heitz et al. [30]. However, if there are more than two channels with different temperature dependencies, with no clear dominant mechanisms, and with possible interaction, the introduction of the semi-empirical ideality factor *η *is a simple approach which can significantly reduce the number of unknowns yet still yield satisfactory results.

**Figure 5:**
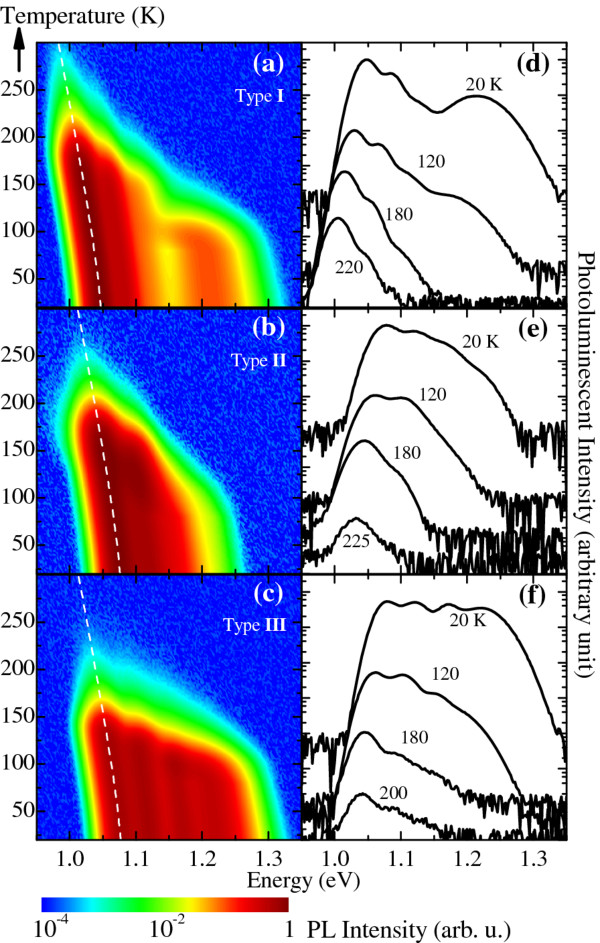
**Simulated PL maps and spectra of chirped QDM bilayers**. Simulated PL maps *I*(*E*,*T*) of (**a**) type-I, (**b**) type-II, and (**c**) type-III chirped QDM bilayers using parameters extracted from the measured spectra in Figure 4a,b,c, respectively. Line spectra at selected temperatures from (a), (b), and (c) are shown in (**d**), (**e**), and (**f**) and are meant to model the measured results in Figure 4d,e,f, respectively. Dashed lines in (a) to (c) are bandgap variations with temperature according to Varshni's equation with bulk InAs parameters, linearly shifted to match the lowest-energy cQD peaks. Spectra in (d) to (f) are offset for clarity.

The simulated PL intensity maps *I*(*E*,*T*) following the approach above and using the parameters extracted from Figure 4a,b,c as summarized in Table 1 are correspondingly shown in Figure 5a,b,c for type I to III chirped QDM bilayers, respectively. Line spectra of Figure 5a,b,c at selected temperatures are shown alongside in Figure 5d,e,f. A white noise with intensity 4 orders of magnitude below the maximum PL peak is added to represent the noise floor of our experimental setup. This aids visual comparisons between the simulated line spectra in Figure 5d,e,f and the measured line spectra in Figure 4d,e,f. The agreements are satisfactory despite the simplicity of the approach and the fact that no moving average algorithms are applied. The latter is required to take into account the effects of finite slit widths of the monochromator and would smoothen the spectra's rising and falling edges as well as all peaks and troughs. While this can improve the qualitative agreements even further, it affects the accuracy of the peak energy positions. Moving-averaged algorithm is thus not carried out to maintain the integrity of Varshni's shift as seen by the dashed lines in Figure 5a,b,c which accurately explain the experimental peak energy shifts of the lowest-energy cQD peaks of the three cases.

**Table 1:** Simulation parameters for the PL maps and spectra of chirped QDM bilayers

		Type I - straddled				Type II - staggered				Type III - broken-gap		
	***I_i_***	***E_i_***	**FWHM**	***η_i_***	***I_i_***	***E_i_***	**FWHM**	***η_i_***	***I_i_***	***E_i_***	**FWHM**	***η_i_***

		**(eV)**	**(meV)**			**(eV)**	**(meV)**			**(eV)**	**(meV)**	

QDM_2_												

sQD_2_	0.071	1.114*	51.8*	1.1-1.9	0.044	1.213*	49.5*	1-1.8	0.7	1.220*	65.9*	1.0-2

cQD_2_	0.358	1.085	28.3	1.0-1.6	0.591	1.120	40.0	1-1.4	0.7	1.170	33.0	1.1-2

QDM_1_												

sQD_1_	0.094	1.214*	77.7*	1.1-1.9	0.315	1.160*	53.0*	1-1.8	1.0	1.121	42.4	1.1-2

cQD_1_	1.000	1.048	30.6	1.0-1.6	1.000	1.077	40.0	1-1.4	1.0	1.078	33.0	1.1-2

20-K peak energy position *E_i_*, relative intensity *I_i_*, and FWHM of cQDs and sQDs ensembles of types I, II, and III chirped QDM bilayers extracted from Figure 4a,b,c, respectively. The ideality factor *η*i varies linearly with temperature from the lower limit value at 20 K to the upper limit value at 300 K. Subscripts 1 and 2 represent the lower and upper QDM layers, respectively. *E_i_*'s temperature dependency follows Varshni's equation unless marked by an asterisk where it instead follows the sigmoidal behavior. FWHM is assumed constant unless marked by an asterisk where it follows the anomalous temperature behavior. The FWHM is related to the standard deviation of the Gaussian distribution or the broadening parameter Γ through the relationship: FWHM (meV) = 1,665.11 × Γ.

In order for chirped InGaAs QDM structures to achieve broadband NIR performance at room temperature, a few design rules should be followed. One, the low-energy emission boundary should be red-shifted without causing intensity drops at intermediate wavelength. This can be achieved by introducing an additional QDM layer with nominally the same *x*/*y*/*z *but using a strain-reducing capping layer instead of GaAs. Nishi et al., for example, shows that replacing GaAs capping with 7-nm In_0.2_Ga_0.8_As redshifts the spectrum by as much as 140 meV [43]. This third QDM layer should be the bottom layer to minimize reabsorption.

Two, the high-energy portion of the spectra should require a greater thermal energy to quench. This can be achieved by changing the barrier material and/or removing the WL. Sanguinetti et al. compared the effect of thermal quenching of luminescence between QDs grown by standard SK epitaxy, with WL, and droplet epitaxy (DE), without WL, and reported a much smaller intensity reduction of the latter due to the absence of WL [44]. If such strategy is employed in our design, and provided no other NRR mechanisms are introduced by the low-temperature growth typical of DE, *E_A _*should increase by approximately 100 meV (the difference between bulk GaAs bandgap and *E_WL_*). Alternatively, or additionally, *E_A _*can be increased by sandwiching the active QDM structures between AlGaAs/GaAs superlattices, but doing so may affect peak energy positions [45]. The high-energy portion of the spectra should be narrow to fully benefit from this design rule; *z *should thus be appropriately thick in proportion to the thicknesses of *x *and *y*. If cQDs and sQDs of all layers are narrow (< 45 meV), the temperature dependencies of all energy peaks will follow Varshni's equation; the low-temperature FWHM and the PL profile will thus be maintained.

Not all lateral QDMs reported to date are compatible with the designs proposed in Figure 3 since at least two different QD ensembles are required. This rules out the essentially one-QDM ensembles grown on superlattice [19] or on nanoholes created by *in situ *etching [18] or by GaAs capping of InAs QDs under As_2 _[23]. For QDMs regrown on GaAs nanoring template [46] formed by nanodrilling [47], however, the designs are applicable since the dots inside and outside the rings are of different sizes. It is interesting to note that though the quad QDMs grown by DE on GaAs nanomounds [20] do have two different QD ensembles, the central QDs are GaAs without barriers. They thus emit at bulk values and are incompatible with the designs. If grown on AlGaAs, however, the emissions from DE GaAs cQDs and SK InGaAs sQDs can be combined to yield a broader bandwidth reported here.

## Conclusions

Chirped bilayer structures employing lateral InGaAs QDMs as active layers are proposed. Ground state emissions from cQDs and sQDs in the bilayer are combined in three basic configurations - straddled, staggered, and broken-gap - to yield multi-GS and broadband spectra. A non-optimized, 170-meV FWHM chirped QDM bilayer is demonstrated, establishing lateral QDMs as a promising broadband NIR material as the bandwidth can be further broadened simply by increasing the stack number and chirping each additional layer with different *x*, *y*, or *z *or replacing the GaAs cap layer with a strain-reducing InGaAs layer. By keeping *x *constant and varying *y *and *z*, we demonstrated the three basic chirping configurations. The temperature-dependent behaviors of the PL spectra of the chirped QDM bilayers are well described in the context of inhomogeneous broadening of constituent QDs and carrier loss to NRR centers via thermally-activated channels. The ideality factor *η *introduced in the intensity reduction term implies a temperature-dependent effective potential barrier, indicating the presence of loss mechanisms/channels in addition to thermionic emission from QDMs to the WL.

## Abbreviations

AFM: Atomic force microscopy; cQD: Central quantum dots; DE: Droplet epitaxy; FWHM: Full-width at half-maximum; GS: Ground state; MBE: Molecular beam epitaxy; ML: Monolayer; NIR: Near infrared; NRR: Non-radiative recombination; PL: Photoluminescence; QD: Quantum dot; QDM: Quantum dot molecule; SK: Stranski-Krastanow; SLD: Superluminescent diodes; sQD: Satellite quantum dots; WL: Wetting layer

## Competing interests

The authors declare that they have no competing interests.

## Authors' contributions

NP grew the samples and measured the luminescence spectra. SP provided technical and managerial supports and supervised the group. SK conceived the study, designed and supervised the experiments, performed the simulation, and wrote the manuscript. All authors read and approved the final manuscript.
